# 10-Formyl-2,4,6,8,12-penta­nitro-2,4,6,8,10,12-hexa­azatetra­cyclo­[5.5.0.0^5,9^.0^3,11^]dodecane acetone solvate

**DOI:** 10.1107/S1600536810002813

**Published:** 2010-01-27

**Authors:** Huaxiong Chen, Lijie Li, Sufen Liu, Shusen Chen, Shaohua Jin

**Affiliations:** a School of Materials Science and Engineering, Beijing Institute of Technology, Beijing 100081, People’s Republic of China; b Gansu Yinguang Chemical Industry Group Co. Ltd, Gansu 730900, People’s Republic of China

## Abstract

The title compound, C_7_H_7_N_11_O_11_·C_3_H_6_O, consisting of one mol­ecule of 10-formyl-2,4,6,8,12-penta­nitro-2,4,6,8,10,12-hexa­azatetra­cyclo­[5.5.0.0^5,9^.0^3,11^]dodecane (penta­nitro­mono­form­yl­hexa­aza­isowurtzitane, PNMFIW) and one acetone solvent mol­ecule, is a member of the caged hexa­azaisowurtzitane family. PNMFIW has a cage structure which is constructed from one six-membered and two five-membered rings which are linked by a C—C bond, thus creating two seven-membered rings. In the PNMFIW mol­ecule, one formyl group is bonded to the N heteroatom of the six-membered cycle, and five nitro groups are appended to other five N heteroatom of the caged structure. The acetone solvent mol­ecule is arranged beside a five-membered plane of PNMFIW with an O atom and an H atom close (with respect to the sum of the van der Waals radii) to the neighbouring nitro O atom [O⋯O = 2.957 (3) and 2.852 (3) Å; O⋯ H = 2.692 (2), 2.526 (3) and 2.432 (3) Å].

## Related literature

For the synthesis see: Golfier *et al.* (1998 ▶); Liu *et al.* (2006 ▶); Ou *et al.* (2000 ▶). For structures with similar properties, see: Chen *et al.* (2010 ▶); Jin *et al.* (2009 ▶); Lu *et al.* (2004 ▶). 
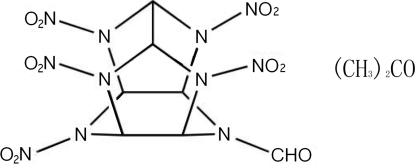


## Experimental

### 

#### Crystal data

C_7_H_7_N_11_O_11_·C_3_H_6_O
*M* *_r_* = 479.31
Monoclinic, 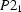

*a* = 10.432 (3) Å
*b* = 7.9230 (19) Å
*c* = 12.191 (3) Å
β = 113.493 (2)°
*V* = 924.1 (4) Å^3^
*Z* = 2
Mo *K*α radiation
μ = 0.16 mm^−1^
*T* = 93 K
0.60 × 0.27 × 0.17 mm

#### Data collection

Rigaku Saturn724+ diffractometer
7388 measured reflections
2257 independent reflections
2056 reflections with *I* > 2σ(*I*)
*R*_int_ = 0.033

#### Refinement

*R*[*F* ^2^ > 2σ(*F* ^2^)] = 0.035
*wR*(*F* ^2^) = 0.066
*S* = 1.00
2257 reflections
301 parameters
1 restraint
H-atom parameters constrained
Δρ_max_ = 0.37 e Å^−3^
Δρ_min_ = −0.25 e Å^−3^

### 

Data collection: *CrystalClear* (Rigaku, 2008 ▶); cell refinement: *CrystalClear*; data reduction: *CrystalClear*; program(s) used to solve structure: *CrystalClear*; program(s) used to refine structure: *SHELXL97* (Sheldrick, 2008 ▶); molecular graphics: *SHELXTL* (Sheldrick, 2008 ▶); software used to prepare material for publication: *SHELXL97*.

## Supplementary Material

Crystal structure: contains datablocks I, global. DOI: 10.1107/S1600536810002813/bg2324sup1.cif

Structure factors: contains datablocks I. DOI: 10.1107/S1600536810002813/bg2324Isup2.hkl

Additional supplementary materials:  crystallographic information; 3D view; checkCIF report

## Figures and Tables

**Fig. 1.**
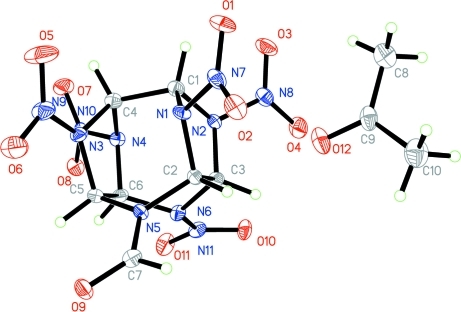
Molecular structure of PNMFIW-acetone with labelling and displacement ellipsoids drawn at the 30% probability level.

## supplementary crystallographic information

### Comment

Title compound is a solvate of PNMFIW (I) with acetone. PNMFIW was even known as a by-product in the synthesis of hexanitrohexaazawurtzitane (HNIW) (Golfier *et al.*, 1998; Liu *et al.*,2006). We determined the single structures of PNMFIW (Jin *et al.*, 2009) and the analog of TNDFIW (Chen *et al.*, 2010) not long ago, further the titled solvate of PNMFIW was obtained by crystallization in acetone solvent.

Figure 1 shows the molecular structure of PNMFIW solvate with acetone. The caged structure of compound (I) is constructed from one six-membered and two five-membered rings which are closed by the C1—C4 bond, thus creating two seven-membered rings. The six-membered pyrazine ring in compound (I) is shaped like a boat, while more stable conformation of six-membered ringis chair form. The two five-membered rings are also non-planar, being characterized by the torsion angles of two five-membered rings. Four nitro groups are appended to the four nitrogen atoms of the two five-membered rings, while a nitro group and a formyl are attached to the two nitrogen atoms of the six-membered ring respectively. It was observed that the acetone molecule is arranged beside a five-membered plane of compound (I) molecule and the skeleton of acetone molecule is nearly perpendicular to the five-membered plane with oxygen atom and a hydrogen atom of acetone significantly closed to the neighbouring nitro oxygen atom of compound (I). The inter molecular distance of O12 of acetone to C2, C3, O2 and O4 are 2.9378 (5), 2.9811 (3), 2.8523 (4) and 2.9567 angstrom much less than the sum of the van der Waals radii respectively,the closed intermolecular contact different from those of the common organic complex (Lu *et al.*, 2004) results the high denstity of 1.723 g/cm^3^.

### Experimental

Fuming sulfuric acid was slowly added into fuming nitric acid in a three-neck flask with stirring. After the solution of mixed acids was heated to 60 ^0^C, tetraacetyldiformylhexaazaisowurtzitane (10 g) was added, and then the temperature was elevated to 65 ^0^C. The solution was maintained at 65 ^0^C for 12 h; thereafter the solution was poured into ice-water. The precipitated solid was filtered off, washed with water and then dried. The obtained solid was a mixture of polynitrohexaazaisowurtzitane derivatives with different number of nitro substitutes. Pure PNMFIW was obtained through a silica column chromatography with hexane/acetyl acetate (6/4 by volume) as mobile phase at room temperature (25 ^0^C).

Pure PNMFIW was dissolved in solvent of acetone, and then the resulted solution was placed in ambient condition (288–293 K). About a week later, single crystals was obtained by controlling the evaporation of solvent. The crystal structure of PNMFIW solvate was determined by single-crystal X-ray diffraction.

### Refinement

All non-hydrogen atoms were obtained using the direct methods. The hydrogen atom were placed geometrically and treated by a constrained refinement. The distances of methane and methene are 1.000 A, and the distance of carbonyl is 0.950 A. The *U*_eq_ of H is assigned 1.2 time *U*_eq_ of C linked.

### Crystal data

**Table:**

C_7_H_7_N_11_O_11_·C_3_H_6_O	*F*(000) = 492
*M**_r_* = 479.31	*D*_x_ = 1.723 Mg m^−^^3^
Monoclinic, *P*2_1_	Mo *K*α radiation, λ = 0.71073 Å
Hall symbol: P 2yb	Cell parameters from 3051 reflections
*a* = 10.432 (3) Å	θ = 3.2–27.5°
*b* = 7.9230 (19) Å	µ = 0.16 mm^−^^1^
*c* = 12.191 (3) Å	*T* = 93 K
β = 113.493 (2)°	Prism, colorless
*V* = 924.1 (4) Å^3^	0.60 × 0.27 × 0.17 mm
*Z* = 2	

### Data collection

**Table:**

Rigaku Saturn724+ diffractometer	2056 reflections with *I* > 2σ(*I*)
Radiation source: Rotating Anode	*R*_int_ = 0.033
graphite	θ_max_ = 27.5°, θ_min_ = 3.2°
Detector resolution: 28.5714 pixels mm^-1^	*h* = −12→13
multi–scan	*k* = −10→10
7388 measured reflections	*l* = −15→13
2257 independent reflections	

### Refinement

**Table:**

Refinement on *F*^2^	Secondary atom site location: difference Fourier map
Least-squares matrix: full	Hydrogen site location: inferred from neighbouring sites
*R*[*F*^2^ > 2σ(*F*^2^)] = 0.035	H-atom parameters constrained
*wR*(*F*^2^) = 0.066	*w* = 1/[σ^2^(*F*_o_^2^) + (0.0252*P*)^2^ + 0.266*P*] where *P* = (*F*_o_^2^ + 2*F*_c_^2^)/3
*S* = 1.00	(Δ/σ)_max_ = 0.001
2257 reflections	Δρ_max_ = 0.37 e Å^−^^3^
301 parameters	Δρ_min_ = −0.25 e Å^−^^3^
1 restraint	Extinction correction: *SHELXL97* (Sheldrick, 2008), Fc^*^=kFc[1+0.001xFc^2^λ^3^/sin(2θ)]^-1/4^
Primary atom site location: structure-invariant direct methods	Extinction coefficient: 0.0080 (18)

### Special details

**Table:**

Geometry. All e.s.d.'s (except the e.s.d. in the dihedral angle between two l.s. planes) are estimated using the full covariance matrix. The cell e.s.d.'s are taken into account individually in the estimation of e.s.d.'s in distances, angles and torsion angles; correlations between e.s.d.'s in cell parameters are only used when they are defined by crystal symmetry. An approximate (isotropic) treatment of cell e.s.d.'s is used for estimating e.s.d.'s involving l.s. planes.
Refinement. Since the absolute configuration for the compound could not reliably be determined from Mo *K*α data, the Friedel equivalents were merged before the final cycles of refinement. Refinement of *F*^2^ against ALL reflections. The weighted *R*-factor *wR* and goodness of fit *S* are based on *F*^2^, conventional *R*-factors *R* are based on *F*, with *F* set to zero for negative *F*^2^. The threshold expression of *F*^2^ > σ(*F*^2^) is used only for calculating *R*-factors(gt) *etc*. and is not relevant to the choice of reflections for refinement. *R*-factors based on *F*^2^ are statistically about twice as large as those based on *F*, and *R*- factors based on ALL data will be even larger.

### Fractional atomic coordinates and isotropic or equivalent isotropic displacement parameters (Å^2^)

**Table:**

	*x*	*y*	*z*	*U*_iso_*/*U*_eq_	
O1	0.5840 (2)	0.7433 (3)	0.04548 (16)	0.0252 (5)	
O2	0.6865 (2)	0.8921 (2)	0.20784 (17)	0.0208 (4)	
O3	0.7530 (2)	0.3757 (3)	0.03918 (17)	0.0271 (5)	
O4	0.9429 (2)	0.3367 (3)	0.19951 (17)	0.0250 (5)	
O5	0.2870 (2)	0.5826 (3)	0.15535 (18)	0.0406 (7)	
O6	0.3470 (2)	0.6539 (3)	0.34336 (18)	0.0331 (6)	
O7	0.39153 (19)	0.0497 (3)	0.18497 (17)	0.0250 (5)	
O8	0.54005 (18)	−0.0268 (2)	0.36166 (16)	0.0192 (4)	
O9	0.64166 (18)	0.7130 (3)	0.58913 (16)	0.0189 (4)	
O10	0.99291 (18)	0.1958 (3)	0.44466 (16)	0.0211 (4)	
O11	0.85172 (19)	0.0667 (3)	0.50810 (17)	0.0256 (5)	
O12	0.9308 (2)	0.7045 (3)	0.23532 (19)	0.0271 (5)	
N1	0.6275 (2)	0.6249 (3)	0.22275 (18)	0.0140 (5)	
N2	0.7407 (2)	0.3766 (3)	0.21929 (18)	0.0136 (5)	
N3	0.4655 (2)	0.4602 (3)	0.29591 (18)	0.0146 (5)	
N4	0.5722 (2)	0.2200 (3)	0.28704 (18)	0.0134 (5)	
N5	0.6735 (2)	0.5978 (3)	0.43095 (17)	0.0120 (4)	
N6	0.7940 (2)	0.3236 (3)	0.42695 (18)	0.0135 (5)	
N7	0.6364 (2)	0.7627 (3)	0.1538 (2)	0.0180 (5)	
N8	0.8190 (2)	0.3654 (3)	0.1471 (2)	0.0188 (5)	
N9	0.3605 (2)	0.5725 (3)	0.2628 (2)	0.0232 (5)	
N10	0.4930 (2)	0.0712 (3)	0.27833 (19)	0.0158 (5)	
N11	0.8852 (2)	0.1841 (3)	0.46067 (19)	0.0171 (5)	
C1	0.6046 (3)	0.4582 (3)	0.1680 (2)	0.0152 (5)	
H1	0.5683	0.4648	0.0788	0.018*	
C2	0.7369 (3)	0.6089 (3)	0.3454 (2)	0.0123 (5)	
H2	0.8035	0.7059	0.3646	0.015*	
C3	0.8126 (3)	0.4392 (3)	0.3415 (2)	0.0146 (5)	
H3	0.9141	0.4587	0.3609	0.018*	
C4	0.4998 (3)	0.3609 (3)	0.2111 (2)	0.0155 (6)	
H4	0.4139	0.3231	0.1420	0.019*	
C5	0.5788 (2)	0.4581 (3)	0.4144 (2)	0.0129 (5)	
H5	0.5417	0.4560	0.4784	0.015*	
C6	0.6499 (3)	0.2865 (3)	0.4086 (2)	0.0145 (6)	
H6	0.6463	0.2060	0.4705	0.017*	
C7	0.6967 (3)	0.7161 (3)	0.5185 (2)	0.0147 (5)	
H7	0.7595	0.8058	0.5242	0.018*	
C8	0.9431 (3)	0.7608 (5)	0.0488 (3)	0.0360 (8)	
H8A	0.8787	0.6650	0.0214	0.043*	
H8B	1.0279	0.7365	0.0355	0.043*	
H8C	0.8980	0.8622	0.0040	0.043*	
C9	0.9808 (3)	0.7892 (4)	0.1793 (3)	0.0217 (6)	
C10	1.0817 (3)	0.9294 (4)	0.2373 (3)	0.0312 (7)	
H10A	1.0981	0.9365	0.3221	0.037*	
H10B	1.0427	1.0364	0.1977	0.037*	
H10C	1.1703	0.9071	0.2299	0.037*	

### Atomic displacement parameters (Å^2^)

**Table:**

	*U*^11^	*U*^22^	*U*^33^	*U*^12^	*U*^13^	*U*^23^
O1	0.0340 (12)	0.0276 (12)	0.0130 (9)	0.0047 (10)	0.0083 (8)	0.0063 (9)
O2	0.0290 (11)	0.0116 (10)	0.0263 (10)	0.0013 (8)	0.0157 (9)	0.0005 (9)
O3	0.0319 (11)	0.0378 (13)	0.0151 (9)	0.0058 (11)	0.0130 (8)	0.0002 (10)
O4	0.0212 (10)	0.0296 (13)	0.0283 (11)	0.0079 (10)	0.0144 (9)	0.0010 (9)
O5	0.0390 (13)	0.0483 (17)	0.0197 (11)	0.0220 (13)	−0.0038 (9)	−0.0022 (11)
O6	0.0302 (12)	0.0425 (15)	0.0235 (11)	0.0185 (10)	0.0075 (9)	−0.0069 (11)
O7	0.0249 (10)	0.0262 (12)	0.0188 (10)	−0.0115 (9)	0.0035 (8)	−0.0052 (9)
O8	0.0236 (10)	0.0142 (11)	0.0215 (10)	0.0010 (8)	0.0109 (8)	0.0034 (8)
O9	0.0222 (10)	0.0207 (10)	0.0183 (9)	−0.0016 (9)	0.0127 (8)	−0.0022 (8)
O10	0.0135 (9)	0.0226 (11)	0.0288 (11)	0.0033 (9)	0.0101 (8)	0.0046 (9)
O11	0.0216 (10)	0.0167 (11)	0.0359 (12)	0.0008 (9)	0.0088 (9)	0.0115 (10)
O12	0.0294 (11)	0.0256 (12)	0.0345 (12)	0.0029 (10)	0.0215 (9)	0.0088 (10)
N1	0.0181 (11)	0.0126 (11)	0.0108 (10)	0.0006 (9)	0.0054 (8)	0.0037 (9)
N2	0.0145 (10)	0.0162 (11)	0.0122 (10)	0.0003 (9)	0.0075 (8)	−0.0009 (9)
N3	0.0126 (10)	0.0175 (12)	0.0123 (10)	0.0033 (9)	0.0033 (8)	−0.0003 (9)
N4	0.0157 (10)	0.0096 (11)	0.0140 (11)	−0.0030 (9)	0.0051 (8)	−0.0008 (9)
N5	0.0146 (10)	0.0119 (11)	0.0113 (10)	−0.0007 (9)	0.0070 (8)	−0.0008 (9)
N6	0.0119 (10)	0.0116 (11)	0.0157 (11)	0.0042 (9)	0.0040 (8)	0.0035 (9)
N7	0.0212 (12)	0.0178 (13)	0.0188 (12)	0.0064 (10)	0.0120 (10)	0.0069 (11)
N8	0.0223 (12)	0.0176 (12)	0.0199 (12)	0.0035 (11)	0.0122 (10)	−0.0006 (10)
N9	0.0208 (12)	0.0254 (14)	0.0190 (12)	0.0099 (11)	0.0033 (10)	0.0003 (11)
N10	0.0172 (11)	0.0150 (12)	0.0182 (11)	−0.0030 (10)	0.0103 (9)	−0.0032 (10)
N11	0.0155 (11)	0.0144 (12)	0.0181 (11)	0.0030 (10)	0.0032 (9)	0.0006 (10)
C1	0.0174 (13)	0.0147 (14)	0.0143 (12)	0.0014 (12)	0.0071 (10)	−0.0008 (11)
C2	0.0130 (12)	0.0128 (14)	0.0119 (12)	0.0010 (11)	0.0059 (10)	0.0010 (10)
C3	0.0154 (12)	0.0159 (14)	0.0122 (12)	−0.0018 (11)	0.0049 (10)	0.0010 (11)
C4	0.0172 (13)	0.0137 (14)	0.0148 (12)	0.0019 (11)	0.0056 (10)	0.0012 (11)
C5	0.0149 (12)	0.0128 (13)	0.0104 (12)	0.0002 (11)	0.0045 (10)	−0.0003 (10)
C6	0.0139 (12)	0.0155 (14)	0.0120 (12)	−0.0017 (11)	0.0029 (10)	0.0016 (10)
C7	0.0146 (12)	0.0105 (13)	0.0173 (13)	0.0030 (11)	0.0044 (10)	−0.0020 (11)
C8	0.0340 (18)	0.044 (2)	0.0315 (17)	−0.0045 (17)	0.0144 (14)	0.0075 (16)
C9	0.0178 (14)	0.0197 (16)	0.0316 (16)	0.0056 (12)	0.0141 (12)	0.0072 (13)
C10	0.0318 (16)	0.0246 (17)	0.0395 (18)	0.0004 (15)	0.0165 (14)	0.0046 (15)

### Geometric parameters (Å, °)

**Table:**

O1—N7	1.221 (3)	N5—C7	1.368 (3)
O2—N7	1.218 (3)	N5—C5	1.443 (3)
O3—N8	1.220 (3)	N5—C2	1.443 (3)
O4—N8	1.213 (3)	N6—N11	1.409 (3)
O5—N9	1.229 (3)	N6—C3	1.458 (3)
O6—N9	1.229 (3)	N6—C6	1.458 (3)
O7—N10	1.219 (3)	C1—C4	1.589 (4)
O8—N10	1.216 (3)	C1—H1	1.0000
O9—C7	1.211 (3)	C2—C3	1.570 (4)
O10—N11	1.218 (3)	C2—H2	1.0000
O11—N11	1.218 (3)	C3—H3	1.0000
O12—C9	1.213 (3)	C4—H4	1.0000
N1—N7	1.403 (3)	C5—C6	1.564 (4)
N1—C1	1.456 (3)	C5—H5	1.0000
N1—C2	1.481 (3)	C6—H6	1.0000
N2—N8	1.423 (3)	C7—H7	0.9500
N2—C1	1.455 (3)	C8—C9	1.496 (4)
N2—C3	1.462 (3)	C8—H8A	0.9800
N3—N9	1.342 (3)	C8—H8B	0.9800
N3—C4	1.453 (3)	C8—H8C	0.9800
N3—C5	1.457 (3)	C9—C10	1.499 (4)
N4—N10	1.419 (3)	C10—H10A	0.9800
N4—C4	1.455 (3)	C10—H10B	0.9800
N4—C6	1.474 (3)	C10—H10C	0.9800
			
N7—N1—C1	117.98 (19)	C3—C2—H2	110.7
N7—N1—C2	117.6 (2)	N6—C3—N2	110.8 (2)
C1—N1—C2	107.7 (2)	N6—C3—C2	107.71 (19)
N8—N2—C1	117.7 (2)	N2—C3—C2	105.15 (19)
N8—N2—C3	117.61 (19)	N6—C3—H3	111.0
C1—N2—C3	107.70 (19)	N2—C3—H3	111.0
N9—N3—C4	123.2 (2)	C2—C3—H3	111.0
N9—N3—C5	123.3 (2)	N3—C4—N4	100.13 (19)
C4—N3—C5	111.5 (2)	N3—C4—C1	111.7 (2)
N10—N4—C4	116.78 (19)	N4—C4—C1	109.3 (2)
N10—N4—C6	116.27 (19)	N3—C4—H4	111.7
C4—N4—C6	107.7 (2)	N4—C4—H4	111.7
C7—N5—C5	122.2 (2)	C1—C4—H4	111.7
C7—N5—C2	122.2 (2)	N5—C5—N3	111.7 (2)
C5—N5—C2	115.5 (2)	N5—C5—C6	111.27 (18)
N11—N6—C3	115.72 (19)	N3—C5—C6	100.23 (19)
N11—N6—C6	115.0 (2)	N5—C5—H5	111.1
C3—N6—C6	116.11 (19)	N3—C5—H5	111.1
O2—N7—O1	126.9 (2)	C6—C5—H5	111.1
O2—N7—N1	116.6 (2)	N6—C6—N4	110.3 (2)
O1—N7—N1	116.2 (2)	N6—C6—C5	107.2 (2)
O4—N8—O3	127.3 (2)	N4—C6—C5	106.0 (2)
O4—N8—N2	116.1 (2)	N6—C6—H6	111.1
O3—N8—N2	116.5 (2)	N4—C6—H6	111.1
O6—N9—O5	126.6 (2)	C5—C6—H6	111.1
O6—N9—N3	116.6 (2)	O9—C7—N5	123.7 (2)
O5—N9—N3	116.8 (2)	O9—C7—H7	118.2
O8—N10—O7	127.0 (2)	N5—C7—H7	118.2
O8—N10—N4	116.1 (2)	C9—C8—H8A	109.5
O7—N10—N4	116.7 (2)	C9—C8—H8B	109.5
O10—N11—O11	126.3 (2)	H8A—C8—H8B	109.5
O10—N11—N6	116.9 (2)	C9—C8—H8C	109.5
O11—N11—N6	116.7 (2)	H8A—C8—H8C	109.5
N2—C1—N1	104.6 (2)	H8B—C8—H8C	109.5
N2—C1—C4	109.0 (2)	O12—C9—C8	121.7 (3)
N1—C1—C4	107.3 (2)	O12—C9—C10	121.5 (3)
N2—C1—H1	111.9	C8—C9—C10	116.8 (3)
N1—C1—H1	111.9	C9—C10—H10A	109.5
C4—C1—H1	111.9	C9—C10—H10B	109.5
N5—C2—N1	110.14 (19)	H10A—C10—H10B	109.5
N5—C2—C3	110.5 (2)	C9—C10—H10C	109.5
N1—C2—C3	103.72 (19)	H10A—C10—H10C	109.5
N5—C2—H2	110.7	H10B—C10—H10C	109.5
N1—C2—H2	110.7		
			
C1—N1—N7—O2	−163.7 (2)	C1—N2—C3—N6	96.0 (2)
C2—N1—N7—O2	−31.9 (3)	N8—N2—C3—C2	115.7 (2)
C1—N1—N7—O1	21.3 (3)	C1—N2—C3—C2	−20.1 (2)
C2—N1—N7—O1	153.1 (2)	N5—C2—C3—N6	0.0 (3)
C1—N2—N8—O4	162.3 (2)	N1—C2—C3—N6	−118.0 (2)
C3—N2—N8—O4	30.8 (3)	N5—C2—C3—N2	118.2 (2)
C1—N2—N8—O3	−22.0 (3)	N1—C2—C3—N2	0.2 (2)
C3—N2—N8—O3	−153.4 (2)	N9—N3—C4—N4	−157.0 (2)
C4—N3—N9—O6	−176.6 (2)	C5—N3—C4—N4	38.7 (3)
C5—N3—N9—O6	−14.2 (4)	N9—N3—C4—C1	87.3 (3)
C4—N3—N9—O5	3.9 (4)	C5—N3—C4—C1	−76.9 (3)
C5—N3—N9—O5	166.3 (3)	N10—N4—C4—N3	98.0 (2)
C4—N4—N10—O8	−160.9 (2)	C6—N4—C4—N3	−34.9 (2)
C6—N4—N10—O8	−32.0 (3)	N10—N4—C4—C1	−144.5 (2)
C4—N4—N10—O7	24.3 (3)	C6—N4—C4—C1	82.6 (2)
C6—N4—N10—O7	153.2 (2)	N2—C1—C4—N3	109.2 (2)
C3—N6—N11—O10	20.2 (3)	N1—C1—C4—N3	−3.5 (3)
C6—N6—N11—O10	160.0 (2)	N2—C1—C4—N4	−0.7 (3)
C3—N6—N11—O11	−163.0 (2)	N1—C1—C4—N4	−113.4 (2)
C6—N6—N11—O11	−23.3 (3)	C7—N5—C5—N3	119.7 (2)
N8—N2—C1—N1	−103.0 (2)	C2—N5—C5—N3	−57.4 (3)
C3—N2—C1—N1	32.7 (2)	C7—N5—C5—C6	−129.2 (2)
N8—N2—C1—C4	142.5 (2)	C2—N5—C5—C6	53.7 (3)
C3—N2—C1—C4	−81.7 (2)	N9—N3—C5—N5	−72.2 (3)
N7—N1—C1—N2	103.4 (2)	C4—N3—C5—N5	92.0 (2)
C2—N1—C1—N2	−32.6 (2)	N9—N3—C5—C6	169.9 (2)
N7—N1—C1—C4	−140.9 (2)	C4—N3—C5—C6	−25.9 (3)
C2—N1—C1—C4	83.0 (2)	N11—N6—C6—N4	−83.5 (2)
C7—N5—C2—N1	−117.8 (2)	C3—N6—C6—N4	56.1 (3)
C5—N5—C2—N1	59.3 (3)	N11—N6—C6—C5	161.54 (19)
C7—N5—C2—C3	128.1 (2)	C3—N6—C6—C5	−58.9 (3)
C5—N5—C2—C3	−54.7 (3)	N10—N4—C6—N6	131.7 (2)
N7—N1—C2—N5	125.2 (2)	C4—N4—C6—N6	−95.1 (2)
C1—N1—C2—N5	−98.5 (2)	N10—N4—C6—C5	−112.6 (2)
N7—N1—C2—C3	−116.5 (2)	C4—N4—C6—C5	20.6 (3)
C1—N1—C2—C3	19.8 (2)	N5—C5—C6—N6	2.2 (3)
N11—N6—C3—N2	82.5 (2)	N3—C5—C6—N6	120.5 (2)
C6—N6—C3—N2	−56.8 (3)	N5—C5—C6—N4	−115.5 (2)
N11—N6—C3—C2	−163.00 (19)	N3—C5—C6—N4	2.7 (2)
C6—N6—C3—C2	57.7 (3)	C5—N5—C7—O9	0.7 (4)
N8—N2—C3—N6	−128.2 (2)	C2—N5—C7—O9	177.6 (2)
